# Vaccination with virosomally formulated recombinant CyRPA elicits protective antibodies against *Plasmodium falciparum* parasites in preclinical in vitro and in vivo models

**DOI:** 10.1038/s41541-020-0158-9

**Published:** 2020-01-31

**Authors:** Marco Tamborrini, Julia Hauser, Anja Schäfer, Mario Amacker, Paola Favuzza, Kwak Kyungtak, Sylvain Fleury, Gerd Pluschke

**Affiliations:** 1 Swiss Tropical and Public Health Institute, Socinstrasse 57, 4002 Basel, Switzerland; 2 University of Basel, Petersplatz 1, 4001 Basel, Switzerland; 3 Mymetics SA, Route de la Corniche 4, 1066 Epalinges, Switzerland

**Keywords:** Protein vaccines, Malaria

## Abstract

The *Plasmodium falciparum* (*Pf*) cysteine-rich protective antigen (*Pf*CyRPA) has emerged as a promising blood-stage candidate antigen for inclusion into a broadly cross-reactive malaria vaccine. This highly conserved protein among various geographical strains plays a key role in the red blood cell invasion process by *P. falciparum* merozoites, and antibodies against *Pf*CyRPA can efficiently prevent the entry of the malaria parasites into red blood cells. The aim of the present study was to develop a human-compatible formulation of the *Pf*CyRPA vaccine candidate and confirming its activity in preclinical studies. Recombinant *Pf*CyRPA expressed in HEK 293 cells was chemically coupled to phosphoethanolamine and then incorporated into the membrane of unadjuvanted influenza virosomes approved as antigen delivery system for humans. Laboratory animals were immunised with the virosome-based *Pf*CyRPA vaccine to determine its immunogenic properties and in particular, its capacity to elicit parasite binding and growth-inhibitory antibodies. The vaccine elicited in mice and rabbits high titers of *Pf*CyRPA-specific antibodies that bound to the blood-stage parasites. At a concentration of 10 mg/mL, purified total serum IgG from immunised rabbits inhibited parasite growth in vitro by about 80%. Furthermore, in a *P. falciparum* infection mouse model, passive transfer of 10 mg of purified total IgG from *Pf*CyRPA vaccinated rabbits reduced the in vivo parasite load by 77%. Influenza virosomes thus represent a suitable antigen delivery system for the induction of protective antibodies against the recombinant *Pf*CyRPA, designating it as a highly suitable component for inclusion into a multivalent and multi-stage virosomal malaria vaccine.

## Introduction

Malaria is a mosquito-borne disease of the tropics caused by *Plasmodium* parasites. Amongst the five species that can affect humans, *Plasmodium falciparum* is the most virulent, causing most of the malaria-related deaths globally. According to the latest World Malaria Report,^1^ in 2017, there were still 219 million cases of malaria leading to 435,000 deaths. Beside this high biomedical burden, malaria impedes social and economic development in endemic areas.^2^ A reduction in the global malaria disease burden has been achieved during the last decade by integrated disease control programs. This gain is at risk by the potential spread of insecticide-resistant mosquitoes and drug-resistant parasites.^1^ Consequently, an effective vaccine would be a complementary tool to reduce the burden of malaria in integrated control programs and could support efforts towards malaria elimination.^3^ Currently no effective malaria vaccine is commercially available, but numerous vaccine design strategies against malaria infection, disease or transmission are being actively pursued, including development of subunit vaccines and whole sporozoite vaccination approaches.^4^

The major symptoms and pathology of malaria are associated with merozoites invasion and replication within red blood cells.^5^ Therefore, a vaccine able to elicit antibodies that effectively prevent the invasion process after release of free merozoites into the bloodstream may reduce parasite burden, disease symptoms and indirectly also transmission. However, extensive allelic polymorphism and redundancy in erythrocyte invasion pathways are limiting strain-transcending neutralisation by traditional merozoite candidate vaccine antigens, such as apical membrane antigen 1 (AMA1) and merozoite surface protein 1 (MSP1).^3,6^ Recently, a few new functionally essential and highly conserved merozoite proteins have emerged as more promising blood-stage candidate vaccine targets,^4^ including the *P. falciparum* cysteine-rich protective antigen (*Pf*CyRPA).^7^

*Pf*CyRPA is a microneme-protein that is essential for merozoite invasion. It fulfils its still unknown role by forming a multi-protein complex at the interface between the merozoite and erythrocyte together with the reticulocyte-binding homolog 5 (*Pf*RH5) and the RH5-interacting protein (*Pf*Ripr).^8,9^ This RH5/Ripr/CyRPA complex plays a central role in the mechanism of *P. falciparum* merozoite invasion as the complex is required for the establishment of tight junctions and the triggering of Ca^2+^ release.^9^ In contrast to the ‘classical’ blood-stage vaccine candidate antigens, *Pf*CyRPA shows very limited genetic diversity and immunogenicity.^7^ *Pf*CyRPA-specific monoclonal antibodies (mAbs) can inhibit parasite growth in vitro by blocking merozoite invasion and show potent in vivo growth-inhibitory activity in *P. falciparum* infected NOD-*scid IL2Rγ*^null^ (NSG) mice engrafted with human erythrocytes.^7,10^ Like *Pf*CyRPA, the two other partners of the invasion complex, *Pf*RH5 and *Pf*Ripr, are vaccine candidates, as they elicit cross-strain invasion-inhibitory antibodies.^8,11–15^ Moreover, anti-*Pf*CyRPA antibodies show: (a) Additive parasite growth-inhibitory activity, when combined with anti-RH5 antibodies both in vitro and in vivo;^16^ and (b) Synergistic in vitro inhibitory activity, when combined with antibodies against some merozoite antigens, such as anti-MSRP5 and anti-RAMA, involved in non-overlapping steps during erythrocyte invasion.^17^ These findings support the inclusion of *Pf*CyRPA into a multi-component subunit malaria vaccine.

Subunit vaccines based on recombinant proteins typically require co-administration with adjuvants, which are either already mixed with the antigens or provided into two separated vials requiring vaccine mixing prior to administration. Also effective particle-based antigen delivery platforms have been developed to achieve adequate immunogenicity. Some antigen delivery systems, like virosomes, can co-deliver immunostimodulators to enhance immunogenicity further.^18^ Preclinical and clinical studies have proven the suitability of influenza derived virosomes as antigen carrier system for malaria-derived peptidomimetics and recombinant proteins.^19^ These virosomes are non-replicating virus-like particles, prepared from the membrane of solubilised influenza virus mixed with synthetic phospholipids and vaccine antigens with or without adjuvants. The hemagglutinin (HA) membrane glycoprotein of the influenza virus harbours T helper cell epitopes that enhance the immune response particularly for small antigens, like peptides. Virosomes loaded with different antigens can be combined to formulate a multi-component subunit vaccine, making them a versatile and modular antigen delivery system with an excellent tolerance and safety profile in children, adults and elderlies, based on experience with the market approved influenza and hepatitis A virosome-based vaccines.^20^

In this study, recombinant *Pf*CyRPA expressed in mammalian cells was lipidated and incorporated into the membrane of human-compatible influenza virosomes. The virosomal formulation without adjuvant and with surface display of recombinant *Pf*CyRPA was used to immunise mice and rabbits to determine its immunogenicity and capacity to elicit parasite binding and inhibitory antibodies capable of blocking erythrocyte invasion in vitro and in vivo.

## Results

### Immunogenicity of *Pf*CyRPA virosomes in mice

**Fig. 1:**
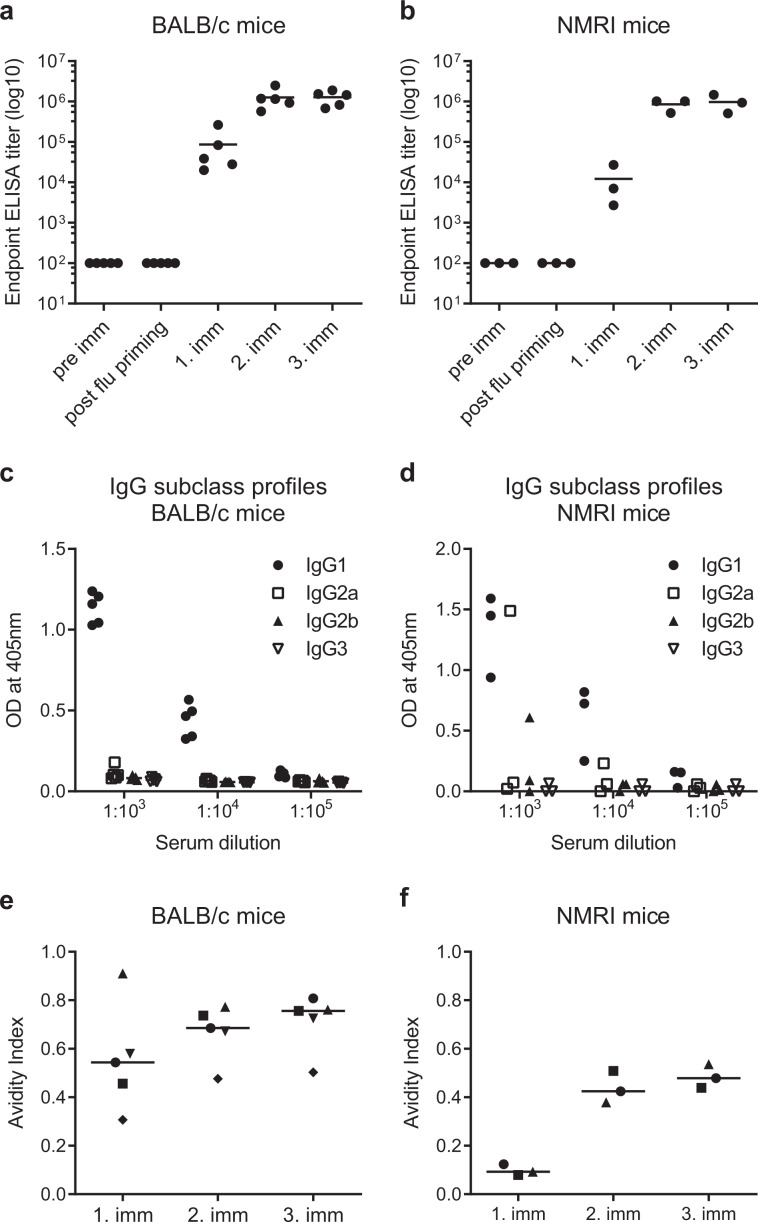
Immunogenicity of *Pf*CyRPA virosomes in mice. Development of anti-*Pf*CyRPA total IgG responses in BALB/c (**a**) and NMRI (**b**) mice after each immunisation. Sera were taken pre-immune (pre imm), after pre-immunisation with inactivated influenza virus (post flu priming) and after the first (1. imm), second (2. imm) and third (3. imm) immunisation with *Pf*CyRPA virosomes. Shown are serum IgG ELISA endpoint titers of individual animals and lines represent the mean. Two independent experiments yielded comparable results; representative data of a single assay is shown. Determination of the IgG subclass profiles in BALB/c (**c**) and NMRI (**d**) mice by ELISA using plates coated with recombinant *Pf*CyRPA. Sera from mice collected after the third immunisation were tested individually at three serum dilutions. Optical density (OD) values form a single experiment are shown; these are representative for the two independent assays. Avidity index for the anti-*Pf*CyRPA IgG responses of BALB/c (**e**) and NMRI (**f**) mice three weeks after the first, second and third immunisation. The avidity index is the NH_4_SCN concentration (M) where 50% of the bound antibodies are eluted. Shown are results from a single experiment obtained with individual sera tested in triplicates and the median (line) for each time point.

Purified recombinant *Pf*CyRPA expressed in mammalian cells was chemically conjugated to phosphoethanolamine and then integrated into the membrane of influenza virosomes. Inbred (BALB/c) and outbred (NMRI) mice were immunised three times with a dose of 20 μg of *Pf*CyRPA virosomes in intervals of three weeks after pre-immunisation with inactivated influenza virus. Pre-immune sera and sera collected after pre-immunisation with inactivated influenza virus showed no reactivity against *Pf*CyRPA in ELISA. The vaccine elicited comparable anti-*Pf*CyRPA IgG titers in the two tested mouse strains. Already one immunisation elicited detectable titers (Fig. 1a, b), and while a second immunisation led to a strong titer increase in all animals, a third immunisation had only a moderate further booster effect. Analyses of the induced *Pf*CyRPA-specific IgG subclass profiles showed a predominance of the IgG1 subclass (Fig. 1c, d). In contrast to BALB/c mice, one outbred NMRI mouse also developed low anti-*Pf*CyRPA IgG2a and IgG2b antibody levels.

To assess avidity maturation of *Pf*CyRPA-specific serum IgG upon repeated immunisation of mice with *Pf*CyRPA virosomes, chaotrope-based avidity measurements were performed. While NMRI mice had low-avidity anti-*Pf*CyRPA IgG antibodies after the first immunisation, respective to BALB/c mice, a five-fold increase of the mean avidity index was observed after the third immunisation (Fig. 1f). In BALB/c mice, the avidity after the first immunisation was already markedly higher than in NMRI mice, and consequently only a gradual increase of the mean avidity index over the course of immunisation was observed (Fig. 1e).

**Fig. 2:**
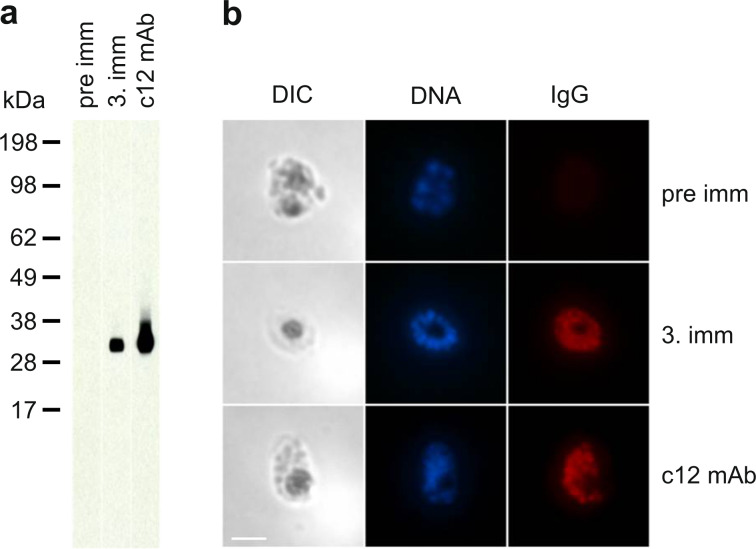
Parasite cross-reactivity of anti-*Pf*CyRPA IgG responses in mice. **a** Pre-immune and immune serum samples of individual mice were tested by immunoblotting analysis with blood-stage parasite lysate. Sera were diluted 1:200 and a representative example obtained with sera from a BALB/c mouse is shown. Blots derived from the same experiment and were processed in parallel. **b** Immune sera of mice were tested for parasite binding in IFA with in vitro cultured *P. falciparum* blood-stage parasites. As a representative example, results obtained with sera from a NMRI mouse are shown. The left panel shows differential interference contrast (DIC), the middle panel DNA staining with DAPI (blue) and the right picture is IgG immunofluorescence staining with Alexa Fluor 568 conjugated secondary antibodies (red). The parasite inhibitory anti-*Pf*CyRPA mAb c12^16^ was used as positive control in both assays. Scale bar, 5 µm.

Induction of *P. falciparum* blood stage cross-reactive IgG upon immunisation with *Pf*CyRPA virosomes was analysed by immunoblotting and immunofluorescence analysis (IFA) with sera collected after the third immunisation. All immune sera of both mouse strains were cross-reactive with *Pf*CyRPA expressed by blood-stage *P. falciparum* schizonts, as detected in immunoblot analyses with parasite lysate. A representative example is shown in Fig. 2a. While immune sera from NMRI mice bound consistently to the blood-stage parasites in IFA (Fig. 2b), immune sera from BALB/c mice yielded weaker and inconsistent immunofluorescence staining signals.

### Immunogenicity of *Pf*CyRPA virosomes in rabbits

**Fig. 3:**
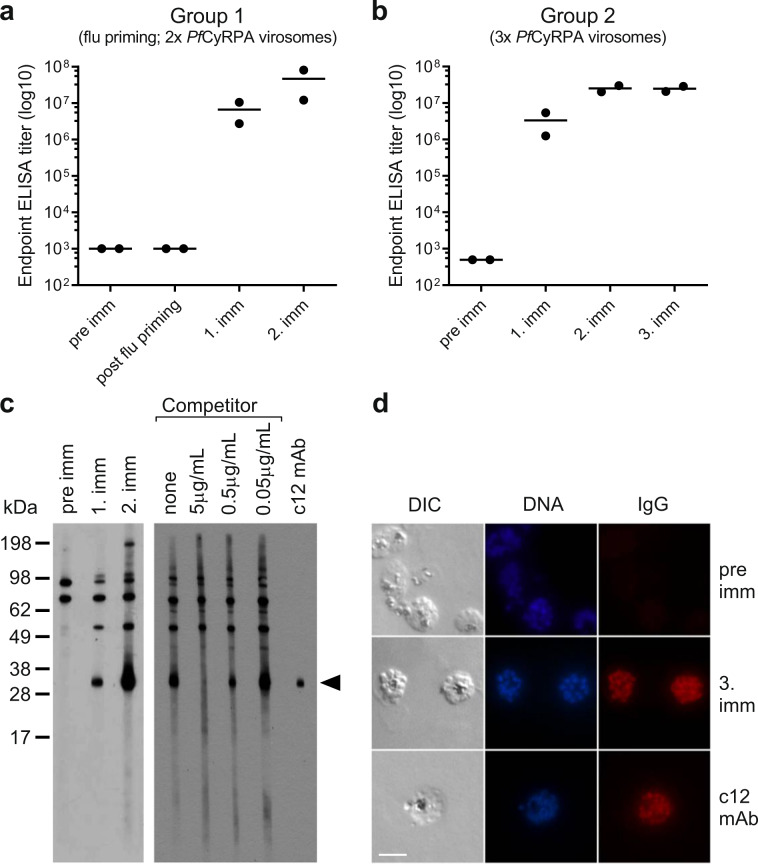
Immunogenicity of virosomally formulated recombinant *Pf*CyRPA in New Zealand rabbits. Groups of two animals were immunised twice (**a**) or three times (**b**) with antigen-loaded virosomes with (**a**) or without (**b**) pre-immunisation with inactivated influenza virus. Shown are serum anti-*Pf*CyRPA IgG ELISA endpoint titers of individual animals and lines represent the mean titer. Two independent experiments yielded comparable results; representative data of a single assay is shown. **c** Immunoblot analysis of rabbit serum samples with blood-stage parasite lysate. Immune serum of a representative rabbit was pre-incubated with or without recombinant *Pf*CyRPA protein competitor and subsequently added to cut strips. Immune serum was used at a dilution of 1:200 and the competitor at a concentration of 5, 0.5 or 0.05 μg/mL. Individual filter stripes of each blot derived from the same experiment and were processed in parallel. **d** Immune sera of rabbits were tested for parasite binding in IFA with in vitro cultured *P. falciparum* blood-stage parasites. Sera were diluted 1:1000 and representative examples are shown. The anti-*Pf*CyRPA neutralising mAb c12 served as positive control. Scale bar, 5 µm.

Preclinical profiling of the virosomal formulation of the recombinant *Pf*CyRPA protein was continued by immunogenicity studies in New Zealand rabbits. While one group of rabbits was immunised twice with *Pf*CyRPA virosomes (40 μg *Pf*CyRPA per dose) after pre-immunisation with inactivated influenza virus, a second group received three immunisations with *Pf*CyRPA virosomes without prior pre-immunisation with inactivated influenza virus. Consistent with the results obtained in mice, all immunised rabbits developed IgG antibodies that interacted with the recombinant *Pf*CyRPA protein in ELISA (Fig. 3a, b). Both immunisation strategies induced an anti-*Pf*CyRPA IgG response already after the first immunisation. Booster effects after the second immunisation were observed in both groups with limited variation in antibody titers. No significant increase in titers resulted from the third immunisation in the second group of animals (Fig. 3b). Pre-immune sera and sera collected after pre-immunisation with inactivated influenza virus showed no cross-reactivity toward *Pf*CyRPA in ELISA.

Immunised rabbits already developed after the first immunisation IgG antibodies that were reactive with *Pf*CyRPA expressed by *P. falciparum* blood-stage schizonts in immunoblot analyses. Immunoblot competition experiments confirmed the binding-specificity, since binding of immune sera to the endogenous protein (band of ∼36 kDa) was inhibited by the recombinant *Pf*CyRPA protein in a concentration dependent manner, whereas background staining of other protein bands observed with pre-immune sera remained unaffected. A representative example is shown in Fig. 3c. Immunisation with *Pf*CyRPA virosomes also induced in all rabbits tested IgG antibodies that reacted with *P. falciparum* blood-stage parasites in IFA. A representative example is shown in Fig. 3d.

### In vitro and in vivo assessment of the parasite inhibitory activity of antibodies generated in rabbits

**Fig. 4:**
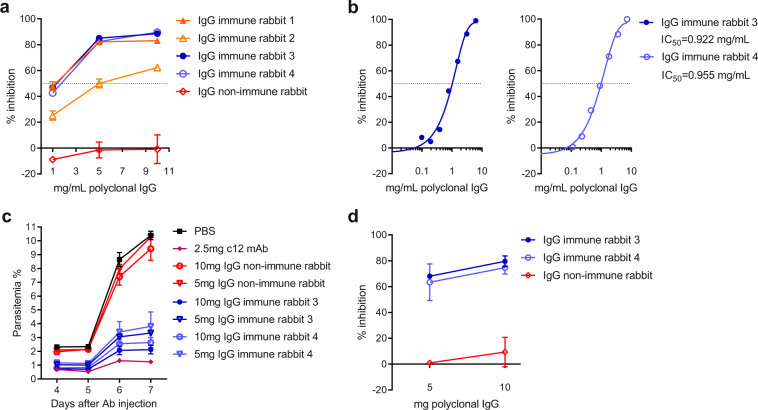
*Pf*CyRPA virosomes elicited polyclonal IgG antibodies with in vitro and in vivo parasite growth-inhibitory activity. **a** Synchronised *P. falciparum* 3D7 blood-stage parasites were cultivated in vitro for 48 h in the presence of different concentrations of purified total serum IgG antibodies from individual rabbits that received two (orange symbols) or three (blue symbols) doses of *Pf*CyRPA virosomes. Purified IgG antibodies from non-immune rabbit sera were used as negative controls. Two independent experiments yielded comparable results; representative data of a single assay performed in triplicate is shown as mean ± standard deviation. **b** In vitro [^3^H]-hypoxanthine incorporation assay to calculate the IC_50_ values for total IgG from individual rabbits that received three doses of *Pf*CyRPA virosomes. Two independent experiments yielded comparable results; data shown as mean of duplicate wells of a single assay is representative for the two independent assays. **c** Passive immunisations of *P. falciparum* infected NSG mice. Humanised NSG mice received either two different doses of purified total serum IgG antibodies formulated in PBS from individual rabbits that received three doses of *Pf*CyRPA virosomes, purified IgG antibodies from non-immune rabbit sera, growth-inhibitory anti-*Pf*CyRPA mAb c12 or PBS. Mice were infected with *P. falciparum* and parasitaemia in peripheral blood was monitored by flow cytometry. **d** Percent parasite growth inhibition six days after infection was calculated against the parasitemia of PBS control mice. Shown are mean values ± standard deviation of two mice per group. The observed parasite growth-inhibitory activities of the anti-*Pf*CyRPA antibodies were confirmed in an independent passive immunoprotection experiment.

Total serum IgG preparations from individual rabbit serum samples taken after the final immunisation with *Pf*CyRPA virosomes were purified to assess the effect of polyclonal anti-*Pf*CyRPA antibodies on the parasite growth. Purified IgG preparations were assessed at concentrations of 10, 5 and 1 mg/mL of total IgG for activity in a single-cycle in vitro parasite growth inhibition assay (GIA) (Fig. 4a). Purified IgG from all immunised rabbits showed substantial dose-dependent parasite in vitro growth-inhibitory activities. In contrast, purified control IgG preparations from non-immunised rabbits showed no significant growth-inhibitory activities against the parasite. While ≥ 80% inhibition was observed for three rabbits at a concentration of 10 mg/mL of total IgG, antibodies from the fourth rabbit (Fig. 4a, rabbit 2 that had received two doses of *Pf*CyRPA virosomes) showed lower parasite growth-inhibitory activity, correlating with an ~6 times lower anti-*Pf*CyRPA IgG ELISA titer (Fig. 3a).

The antibody concentrations giving 50% growth inhibition (IC_50_ values) were determined for IgG preparations from rabbits that received three doses of *Pf*CyRPA virosomes in an [^3^H]-hypoxanthine incorporation assay (Fig. 4b), which is commonly used to measure the activity of antimalarial compounds against intra-erythrocytic forms of *P. falciparum*. The IC_50_ against 3D7 parasites was 0.92 and 0.96 mg/mL of total IgG, respectively (Fig. 4b).

The in vivo parasite inhibitory activity of the generated anti-*Pf*CyRPA antibodies was evaluated in passive immunoprotection experiments using a humanised *P. falciparum* NSG mouse model. In this model, the immunodeficient NSG mice engrafted with human erythrocytes are susceptible to infection by the human *P. falciparum* parasite, allowing the protective efficacy of antibodies specific for *P. falciparum* blood-stage antigens to be tested. Humanised NSG mice were infected intravenously with 3 × 10^7^ parasitized erythrocytes obtained from in vitro cultures of strain Pf3D7^0087/N9^ and the parasitemia evolution was monitored daily starting on day 3 after infection. One day before infection (day 1), groups of mice received a single dose of 10 or 5 mg of purified total serum IgG from individual rabbits that had received three immunisations with *Pf*CyRPA virosomes. Control groups received either purified serum IgG from non-immunised rabbits or PBS without antibodies. In the PBS control group, parasitemia (percentage of the total number of human erythrocytes that were parasitized) reached 10.4 ± 0.5% on day 7 (6 days post infection when the experiment was terminated; Fig. 4c). Passive immunisation with polyclonal anti-*Pf*CyRPA antibodies had a substantial, dose-dependent parasite growth-inhibitory effect. The administration of 10 mg of purified total polyclonal immune rabbit IgG reduced parasite growth by 77.1 ± 4.7% (Fig. 4d). Parasitaemia in these mice increased only marginally, reaching 2.4 ± 0.5% on day 7 after antibody injection (Fig. 4c). The 5 mg dose still reduced parasite growth on average by 65.7 ± 8.7%. Purified control rabbit IgG from non-immune animals showed no significant growth-inhibitory activities.

The decline in the concentration of total rabbit IgG in the circulation of the passively immunised mice was monitored by ELISA analysis using the purified total IgG preparations as references. One day after antibody administration, when mice were infected, the total rabbit IgG concentrations were estimated to be 1.23 ± 0.64 and 0.46 ± 0.16 mg/mL of serum, for the 10 and 5 mg doses, respectively. At the end of the experiment (day 7), rabbit IgG levels had dropped to an estimated 0.29 ± 0.08 and 0.18 ± 0.07 mg/mL, for the 10 and 5 mg doses, respectively.

## Discussion

The objective of the present study was to develop a human-compatible formulation of the *Pf*CyRPA vaccine candidate antigen and to confirm its protective potential in preclinical studies. Protein subunit vaccine approaches typically require co-administration of strong adjuvants or effective antigen delivery systems such as virus-like particles to overcome the poor immunogenicity of recombinant proteins. Here we tested a monovalent candidate malaria vaccine based on the recombinant *Pf*CyRPA target protein displayed on the surface of influenza virosomes, representing virus-like particles approved for use in humans. While in this first study unadjuvanted *Pf*CyRPA influenza virosomes were tested, adjuvanted human-compatible formulations are under development that may offer the advantage to have both antigens and adjuvants bound to the same particle with no free-form adjuvant, improving the immunogenicity and safety profile of the vaccine. The concept of using antigen-loaded influenza virosomes for vaccination against malaria in humans has already been clinically validated with a virosomal formulation incorporating optimised peptidomimetics derived from the circumsporozoite protein (CSP) and the apical membrane antigen 1 (AMA-1).^19^ This bivalent virosomal vaccine candidate demonstrated excellent safety, immunogenicity and pilot efficacy in a Phase 1b trial in African children.^21^ Antigen delivery using influenza virosomes enables the design of multivalent vaccines, as different populations of antigen-loaded virosomes can be combined. This is an important prerequisite, since it is generally assumed that a highly effective malaria subunit vaccine has to target antigens of several developmental stages of the parasite.

*Pf*CyRPA virosomes were immunogenic, leading to seroconversion in all immunised mice and rabbits; two immunisations were enough to elicit maximal anti-*Pf*CyRPA IgG ELISA titers. Results confirmed that pre-immunisation of the rabbits with inactivated influenza virus is not a prerequisite for the induction of antibodies against heterologous antigens on virosomes and that pre-existing influenza immunity does not prevent antibody induction.^22^ IgG avidity maturation, indicative for memory B cell formation, was evidenced by an increase in anti-*Pf*CyRPA antibody avidity over the course of immunisations. Besides antibody fine-specificity and binding avidity, recently also the kinetic parameters for antibody binding to its target emerged as a key indicator of potency for merozoite neutralising antibodies. The ability of recombinant human anti-*Pf*RH5 mAbs generated after vaccination with viral vectored *Pf*RH5 to inhibit parasite growth in vitro correlated with antibody association-rates. Moreover, one anti-*Pf*RH5 mAb without neutralising activity potentiated the activities of other inhibitory antibodies indirectly, by slowing down the invasion time of merozoites and thereby increasing the time window for inhibitory antibodies to act.^23^

The *Pf*CyRPA vaccine-induced parasite-binding IgG antibodies, confirming that virosomes presented the lipidated recombinant *Pf*CyRPA protein in its native conformation to the immune system. After having established that vaccine-induced antibodies bound to the endogenous *Pf*CyRPA protein expressed in the invasive stage of the parasite blood-stage cycle, we conducted in vitro GIAs with blood-stage parasites cultured in human red blood cells. This analysis compares invasion and growth of parasites in the absence and presence of antibodies. Although validation of the antibody-mediated GIA as a correlate of protection in humans has been difficult to establish, this assay has been commonly used to select and prioritise blood-stage vaccine candidates for final preclinical and clinical development. Purified total IgG preparations of all *Pf*CyRPA-immunised rabbits showed substantial and dose‐dependent GIA activities. At a concentration of 5 mg/mL total IgG, 50–85% growth inhibition was observed with all rabbit IgG samples. While the small number of animals per group limits statistical interpretation of the results, higher mean activities were observed in rabbits that received three doses of antigen-loaded virosomes. The mean total IgG concentration giving 50% GIA for rabbits that received three vaccine doses was 0.94 mg/mL total IgG. This value lies within the broad range of GIA IC_50_ values (between 0.38 mg/mL^17^ and 5.07 mg/mL^24^) that have been reported for total IgG generated by immunising rabbits with adjuvanted recombinant *Pf*CyRPA. All three studies used recombinant *Pf*CyRPA proteins produced in HEK 293 cells but the immunisation strategies were not equivalent. In the study of Bustamante et al.,^17^ which reported the lowest GIA IC_50_ value, rabbits were immunised four times with a total amount of 0.3–0.6 mg of purified antigen. The adjuvant used has not been reported. In the trial published by Illingworth et al.,^24^ rabbits were immunised three times and each dose consisted of 100 μg antigen and 200 μL AddaVax™ adjuvant. The differences in the GIA results could thus have arisen from factors such as differences in antigen dose, adjuvant activity, immunisation protocol and GIA assay format.

In malaria vaccine development, the preclinical evaluation of vaccine efficacy in vivo is complicated by the absence of simple non-primate animal models for *P. falciparum*. The development of an improved murine model of malaria using *P. falciparum* competent strains and NSG mice engrafted with human erythrocytes,^25^ which was established and is extensively used for malaria drug development, has opened new possibilities for the preclinical evaluation and prioritisation of candidate vaccines against erythrocytic stages of *P. falciparum*. We adopted the improved *P. falciparum* infection mouse model for the assessment of the efficacy of antibodies against blood-stage antigens in passive immunoprotection experiments.^7^ Antibody transfer experiments in this in vivo model demonstrated dose-dependent and reproducible growth-inhibitory effects of mouse mAbs specific for the blood-stage vaccine candidates *Pf*CyRPA and *Pf*RH5.^7,10,16^ In the present study, humanised NSG mice were passively immunised with purified total IgG from individual rabbits that received three vaccine doses of antigen-loaded virosomes. One day after the antibody transfer, mice were infected with *P. falciparum* 3D7 parasites. Passive transfer of vaccine-induced polyclonal anti-*Pf*CyRPA antibodies did not completely prevent establishment of infection but reduced parasite growth in a dose-dependent manner by about 77% (10 mg dose) and 66% (5 mg dose) on day 6 after infection. The concentration of total rabbit IgG in the circulation of the passively immunised mice that received 10 mg of total IgG, was estimated to be 1.23 mg/mL one day after administration (the day of infection) and 0.29 mg/mL at day 7 (the day of the measured final parasitemia). These concentrations were from the beginning of the parasite challenge much lower than the concentration of IgG in normal rabbit serum, which ranges from 5 to 12 mg/mL. Considering in addition the relatively quick in vivo clearance of the rabbit antibodies, while the parasite keeps replicating, the inhibition of parasite growth is remarkable. The *Pf*CyRPA-specific IgG is expected to represent only a small fraction of the total IgG. In previous passive immunoprotection experiments with parasite inhibitory monoclonal antibodies, administration of 2.5 mg inhibitory anti-*Pf*CyRPA or anti-*Pf*RH5 mAbs was required to achieve a maximal parasitemia reduction of about 90%.^10,16^ An even higher reduction of parasite burden (95% inhibition) was observed in the mouse model when 2.5 mg inhibitory anti-*Pf*CyRPA and 2.5 mg inhibitory anti-*Pf*RH5 mAbs were injected in combination.^16^

Taken together, these results demonstrate that recombinantly expressed *Pf*CyRPA delivered on human-compatible influenza virosomes was immunogenic in preclinical animal models and induced strong growth-inhibitory antibodies against *P. falciparum* blood-stage parasites that were functional both in vitro and in vivo. Recombinant *Pf*CyRPA thus represents a highly suitable highly conserved antigen candidate component for inclusion into a multivalent virosomal malaria vaccine, with excellent cross-strain inhibitory potential.

## Methods

### Recombinant protein production and candidate vaccine formulation

Recombinant *P. falciparum* CyRPA, a histidine-tagged protein comprising residues 29–362 and containing no N-glycosylation sites, was produced and purified as described.^10^ Briefly, synthetically manufactured DNA sequences encoding *Pf*CyRPA were cloned into a pcDNA3.1-based expression vector, which allows secretion of the protein of interest into the cell culture supernatant. Plasmids were amplified by transforming chemically competent *Escherichia coli* bacteria and used for transfection of human embryonic kidney HEK 293 cells. The histidine-tagged protein was purified from concentrated cell culture supernatant by immobilised metal ion affinity chromatography. The purified recombinant *Pf*CyRPA protein was lipidated prior to incorporation into the influenza virosomes membrane derived from the flu strain A/Brisbane/59/2007 (H1N1). In short, purified *Pf*CyRPA in 50 mM HEPES pH 7.4, 142.5 mM NaCl buffer was randomly modified on accessible lysine residues by reacting with a five-fold molar excess of 2-Iminothiolane for 30 min at 4 °C, before addition of the maleimide-derivatized phospholipid N-MCC-DPPE (1,2-Dipalmitoyl-sn-glycero-3-phosphoethanolamine-N-[4-(p-maleinimidomethyl)cyclohexane-carboxamide], Corden Pharma LLC, Switzerland) for 1 h at room temperature. Unused maleimide groups were quenched by the addition of cysteine. SDS-PAGE analysis confirmed the modification of > 95% of the *Pf*CyRPA molecules. Native immunoblot and ELISA analysis confirmed the reactivity of the modified protein with the parasite inhibitory anti-*Pf*CyRPA mAb c12 to the same extend as the unmodified *Pf*CyRPA. The virosomal formulations with the lipidated *Pf*CyRPA were prepared essentially as described.^20,26^ The lipidated *Pf*CyRPA was mixed with the membrane fraction of the inactivated influenza virus strain A/Brisbane/59/2007 (H1N1), solubilized in 100 mM dodecyl octaethylene glycol ether (OEG; Sigma, Switzerland) in 50 mM HEPES pH 7.4, 142.5 mM NaCl buffer, and with synthetic 1,2-dioleoyl-sn-glycero-3-phosphocholine (DOPC; Merck & Cie, Switzerland). The concentrated virosome intermediate mixture with the integrated *Pf*CyRPA protein was obtained after removal of OEG on polystryrene beads (Bio-Beads; Bio-Rad, Switzerland) in a batch chromatography. After determination of the *Pf*CyRPA antigen concentration by ELISA and native immunoblot, the final vaccine was prepared by dilution of the intermediate mixture to the required final antigen concentration in HEPES-NaCl pH 7.4 buffer, followed by a final filtration step on a 0.22 µm PVDF syringe filter.

### Immunogenicity studies in laboratory animals

Mice experiments were carried out in accordance with the national regulations for the protection of animal rights. The protocols were ethically approved by the veterinary office of the county of Basel-City, Switzerland (Permit Numbers: 2375 and 2303). Seven-week-old specific pathogen-free Crl:NMRI(Han) (*n* = 3) and BALB/cAnNCrl (*n* = 5) mice were purchased from Charles River Laboratories (Germany) and used for immunisation studies. Mice were pre-immunised subcutaneously with inactivated influenza virus (equivalence of 1 μg of haemagglutinin from A/Brisbane/59/2007 H1N1) to induce immune responses toward viral membrane proteins for mimicking human pre-existing influenza immunity. Three weeks later, they were immunised subcutaneously with *Pf*CyRPA conjugated to virosomes (20 μg *Pf*CyRPA per dose) in intervals of three weeks (day 0, 21 and 42). Blood was collected before each immunisation and two weeks after the final injection.

New Zealand rabbits were kept, immunised and bled at Kaneka Eurogentec S.A. (Belgium). One group of rabbits (*n* = 2) was immunised twice with *Pf*CyRPA virosomes (40 μg *Pf*CyRPA per dose) by the intramuscular route after pre-immunisation with inactivated influenza virus (equivalence of 10 μg of haemagglutinin). A second group of animals received three immunisations in intervals of at least three weeks without pre-immunisation with inactivated influenza virus. Blood was collected before each immunisation and three weeks after the final injection. Total IgG was purified from rabbit sera using protein A columns (GE Healthcare).

### ELISA

For the detection of CyRPA-specific IgG antibodies in sera, Nunc MaxiSorp^TM^ flat-bottom 96-well ELISA plates were coated with 5 μg/mL of purified recombinant *Pf*CyRPA protein overnight at 4 °C. After blocking and washing, plates were incubated with serial dilutions of mouse or rabbit sera in phosphate buffer saline (PBS) at pH 7.4 for one hour at room temperature. The plates were then washed and incubated with goat anti-mouse (Sigma) or anti-rabbit IgG (Jackson ImmunoResearch) conjugated to horseradish peroxidase (HRP) secondary antibodies for 1 h at room temperature. Tetramethylbenzidine (TMB) was used as substrate (KPL). The reaction was stopped after appropriate time with 0.5 M H_2_SO_4_ and the absorbance was read at 450 nm with the Sunrise absorbance plate reader (Tecan). Data were processed and analysed using GraphPad Prism 7.

The anti-*Pf*CyRPA IgG subclasses were determined by ELISA with alkaline phosphatase-conjugated goat anti-mouse IgG1, IgG2a, IgG2b and IgG3 secondary antibodies (SouthernBiotech).

In avidity ELISA analyses,^27^ mouse serum samples were added to *Pf*CyRPA-coated ELISA plates in triplicates at constant dilutions (approx. halfmax titer). After washing, plates were incubated for 15 min with NH_4_SCN diluted in 0.1 M NaH_2_PO_4_ buffer (pH 6) at the following molarities: 5 M, 4 M, 3 M, 2 M, 1 M, 0.5 M, and 0.25 M. After a wash step, the plates were incubated with goat anti-mouse HRP secondary antibodies for detecting antibodies that remained bound to the antigen and processed as described above. The avidity index corresponds to the NH_4_SCN concentration (M) eluting 50% of the bound antibodies.

### Immunoblotting

Cultured *P. falciparum* 3D7-infected erythrocytes were collected at the schizont stage and treated with 0.06% saponin on ice for 20 min to release the parasites. Washed parasites were resuspended in PBS pH 7.4 and frozen at −80 °C. After thawing, parasite cells were lysed in RIPA buffer (1% NP40, 0.25% DOC, 10% glycerol, 2 mM EDTA, 137 mM NaCl, 20 mM Tris HCl pH 8.0, with a cocktail of protease inhibitors) for 10 min on ice. The lysates were cleared by centrifugation at 15,000 *×* *g* for 10 min and the supernatant kept at −80 °C until use. Parasite lysates were heated to 70 °C for 10 min under non-reducing conditions in LDS sample buffer and resolved on precast 4–12% gradient gels (NuPAGE^TM^ Novex 4–12% Bis–Tris Gel, Invitrogen). Following gel electrophoresis, separated proteins were transferred to nitrocellulose membranes using a dry-blotting system (iBlot, Invitrogen). After blocking, the membranes were incubated with appropriate dilutions of sera or mAbs in PBS for 1 h at room temperature. In competition experiments, primary antibodies were pre-incubated for 30 min with recombinant *Pf*CyRPA protein competitor at a concentration of 5, 0.5 or 0.05 μg/mL. After washing, blots were incubated with secondary HRP-conjugated antibodies and bands were visualised using ECL detection kit (Pierce). Uncropped scans of the blots from Figs 2a and 3c are shown in the Supplementary Figs 1 and 2.

### Indirect immunofluorescence assay

Thin smears of synchronised *P. falciparum* 3D7-infected erythrocytes were fixed in 60% methanol and 40% acetone for 2 min at −20 °C, air-dried and blocked with 3% BSA in PBS. Cells were then incubated with appropriate dilutions of sera or mAbs in PBS for 1 h at room temperature. After washing, cells were incubated with secondary antibodies specific for mouse or rabbit IgG conjugated with Alexa Fluor 568 (Invitrogen). Before the immunoreactivity was analysed with a Leica DM-5000B fluorescence microscope using a 60x oil immersion objective lens, the slides were washed, mounted with ProLong^TM^ Gold antifade reagent containing DAPI (Invitrogen) and covered with a coverslip. Images were processed using Leica Application Suite V4 and Adobe Photoshop CC.

### In vitro growth inhibition assay

In vitro growth inhibition assays with the laboratory-adapted *P. falciparum* 3D7 clone were performed essentially as described.^28^ Synchronised trophozoites were adjusted to 0.5% parasitemia and then incubated for 48 h with various concentrations of purified rabbit IgG in PBS at 1% haematocrit. Each culture was set up in triplicate in 96-well flat-bottomed culture plates. Final parasitemia was quantified by flow cytometry after staining of viable parasites with hydroethidine and inhibition calculated relative to infection control wells containing PBS only. The gating strategy used for parasitemia quantification is shown in Supplementary Fig. 3a.

IC_50_ values for total IgG from *Pf*CyRPA-immunised rabbits were determined in vitro by measuring incorporation of the nucleic acid precursor [^3^H]-hypoxanthine.^29^ Infected red blood cells were exposed to increasing concentrations of purified total rabbit IgG in culture plates. After 48 h of incubation, 0.5 μCi [^3^H]-hypoxanthine was added to each well. Cultures were incubated for further 24 h before they were harvested onto glass-fibre filters and washed with distilled water. The radioactivity was counted using a Betaplate liquid scintillation counter. The results were recorded as counts per minute per well at each IgG concentration and expressed as percentage of the untreated controls. For each individual rabbit a four-parameter sigmoidal dose-response curve was fitted to the relationship between log_10_(antibody concentration) and % inhibition, and then used to interpolate IC_50_ values. Data were processed and analysed using GraphPad Prism 7.

### Passive immunisations of *P. falciparum* infected NSG mice

The in vivo parasite inhibitory activity of the generated polyclonal anti-*Pf*CyRPA antibodies was tested in the *P. falciparum* mouse infection model essentially as described.^7,25^ Six to eight week old female NSG mice (The Jackson Laboratory) were injected almost daily with 0.75 mL of human erythrocytes suspended in RPMI 1640 solution and decomplemented human serum at 50% haematocrit. Eleven days after the start of blood injections, groups of humanised mice (*n* = 2) received a single dose of 10 or 5 mg of purified total serum IgG antibodies by intraperitoneal injection. The following day, mice were infected intravenously with 3 × 10^7^ parasitized erythrocytes obtained from in vitro cultures (strain Pf3D7^0087/N9^). The degrees of engraftment and parasitemia in peripheral blood were measured by flow cytometry using SYTO-16 green fluorescent nucleic acid stain (Invitrogen) and an allophycocyanin-labelled rat anti-mouse erythrocyte TER-119 monoclonal antibody (BD Biosciences). The gating strategy used for parasitemia quantification is shown in Supplementary Fig. 3b.

### Reporting summary

Further information on experimental design is available in the Nature Research Reporting Summary linked to this article.

## Supplementary information

Supplementary Information

Reporting Summary

## Data Availability

The datasets generated during and/or analysed during the current study are available from the corresponding author on reasonable request.
