# Supplementary material for: Vaccination with virosomally formulated recombinant CyRPA elicits protective antibodies against Plasmodium falciparum parasites in preclinical in vitro and in vivo models
Source: NPJ Vaccines. 2020 Jan 31;5:9. doi: 10.1038/s41541-020-0158-9 (PMC6994490; doi:10.1038/s41541-020-0158-9)
Supplement: Supplementary file 1 — Supplementary Information [file 41541_2020_158_MOESM1_ESM.pdf]

Supplementary information

Vaccination with virosomally formulated recombinant CyRPA elicits protective antibodies against *Plasmodium falciparum* parasites in preclinical *in vitro* and *in vivo* models

Marco Tamborrini[1,2]*, Julia Hauser[1,2], Anja Schäfer[1,2], Mario Amacker[3], Paola Favuzza[1,2], Kwak Kyungtak[1,2], Sylvain Fleury[3] and Gerd Pluschke[1,2]

[1]Swiss Tropical and Public Health Institute, Socinstrasse 57, 4002 Basel, Switzerland

[2]University of Basel, Petersplatz 1, 4001 Basel, Switzerland

[3]Mymetics SA, Route de la Corniche 4, 1066 Epalinges, Switzerland

*Correspondence: Marco Tamborrini, Molecular Immunology, Swiss Tropical and Public Health Institute, Socinstrasse 57, 4002 Basel, Switzerland. Phone: + 41 61 284 83 40. E-mail: marco.tamborrini@swisstph.ch

Supplementary Figures

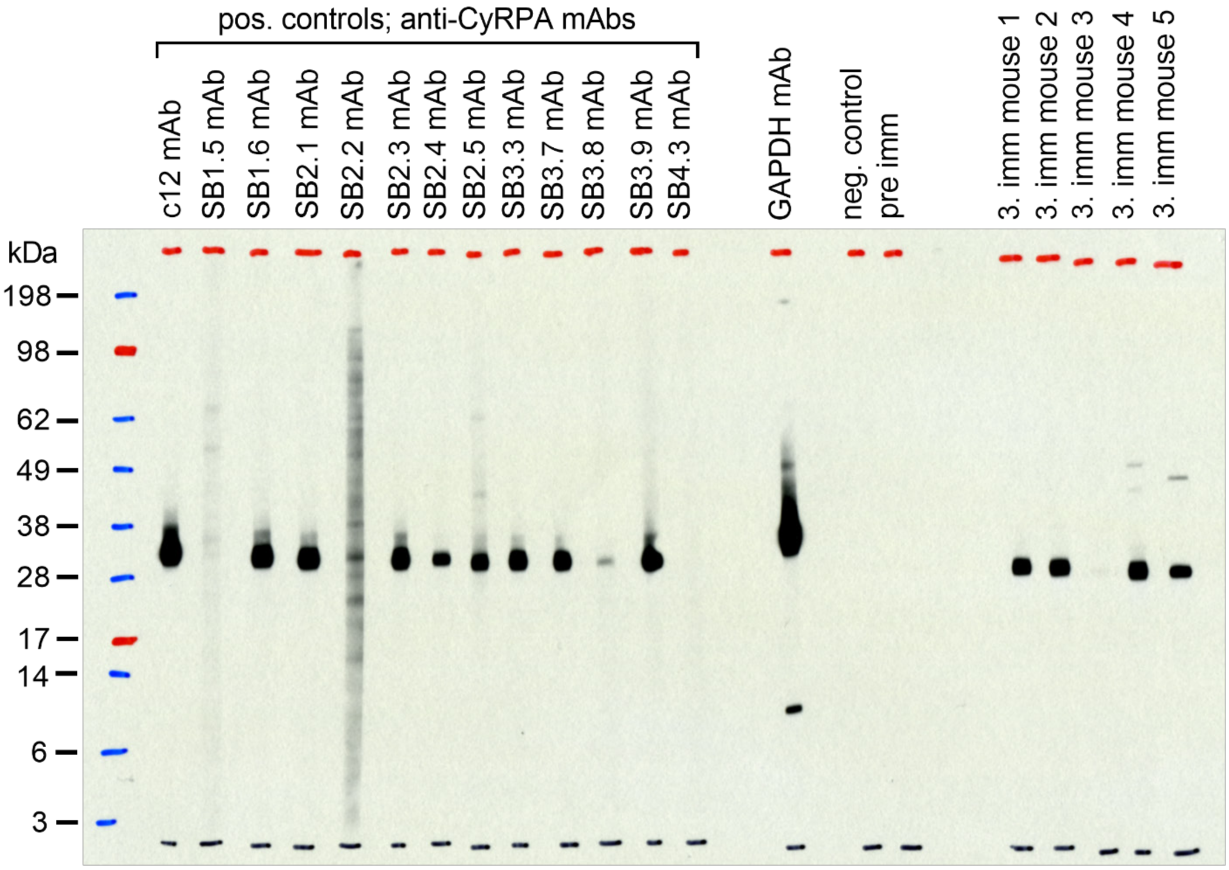

Supplementary Figure 1 Full, uncropped blot from Figure 2a with molecular weight marker.

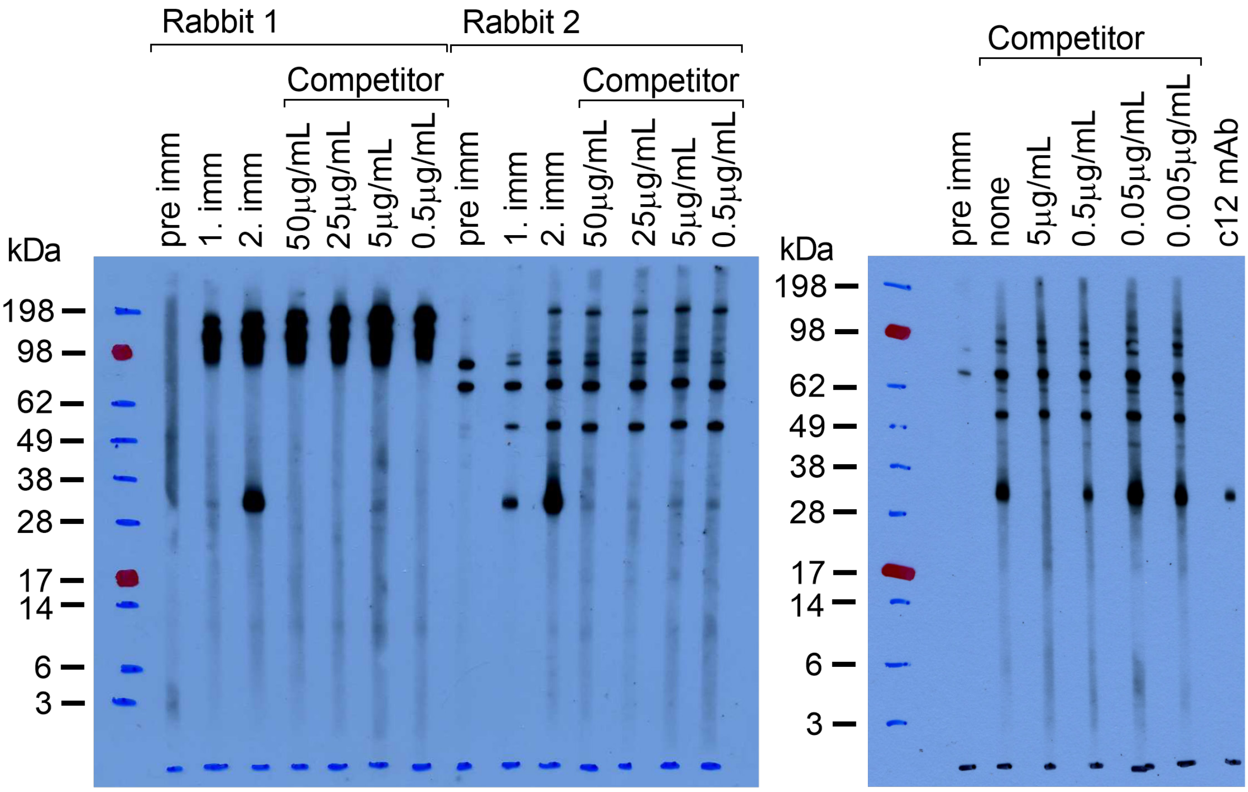

Supplementary Figure 2 Full, uncropped blots from Figure 3c with molecular weight marker.

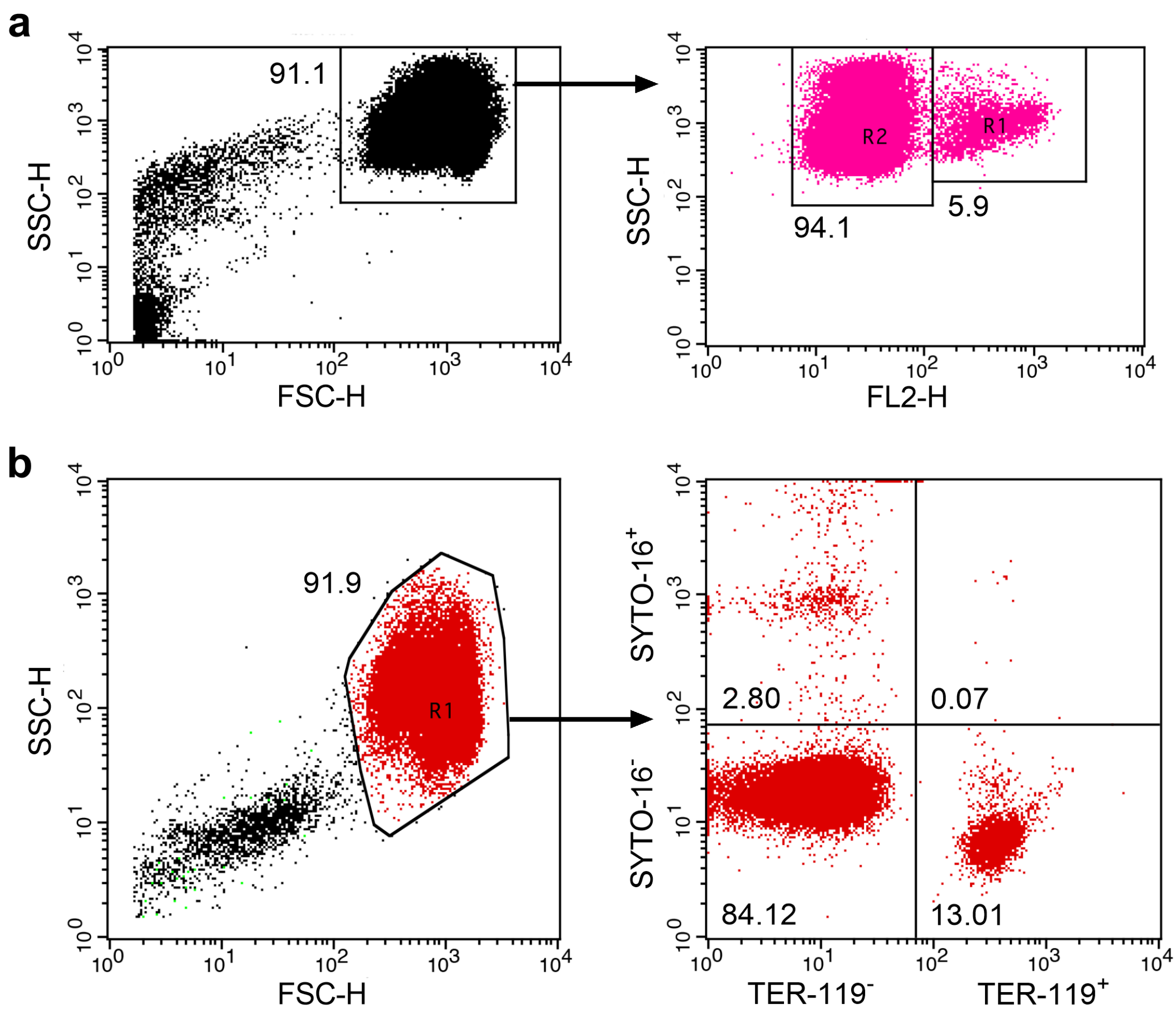

Supplementary Figure 3. Gating strategies used for parasitemia quantification. **a** For the analysis of *in vitro* growth-inhibition assays (Figure 4a), erythrocytes were first gated on the basis of their forward and side scatters and the hydroethidine emission was detected in the appropriate channel by logarithmic amplification. R1= infected human erythrocytes. R2= human erythrocytes. **b** For the analysis of *in vivo* growth-inhibition in the humanized mouse model (Figure 4c and d), erythrocytes of peripheral blood samples were first gated based on morphological criteria (SSC v/s FSC cytogram). In the quadrant dot plot setting, mouse erythrocytes were gated as TER-119$^+$, human erythrocytes as TER-119$^-$ and infected human erythrocytes as TER-119$^-$ and SYTO-16$^+$. R1= erythrocytes.
